# Extract and Active Principal of the Neotropical Vine *Souroubea sympetala* Gilg. Block Fear Memory Reconsolidation

**DOI:** 10.3389/fphar.2019.01496

**Published:** 2019-12-20

**Authors:** Anthony Murkar, Christian Cayer, Jon James, Tony Durst, John T. Arnason, Pablos E. Sanchez-Vindas, M. Otarola Rojas, Zul Merali

**Affiliations:** ^1^The Royal’s Institute of Mental Health Research affiliated with the University of Ottawa , Ottawa, ON, Canada; ^2^School of Psychology, University of Ottawa, Ottawa, ON, Canada; ^3^Centre for Advanced Research in Environmental Genomics, Ottawa-Carleton Institute of Biology, University of Ottawa, Ottawa, ON, Canada; ^4^Department of Cellular and Molecular Medicine, University of Ottawa, Ottawa, ON, Canada; ^5^Department of Chemistry and Biomolecular Sciences, University of Ottawa, Ottawa, ON, Canada; ^6^Herbario Juvenal Valerio Rodriguez, Universidad Nacional, Heredia, Costa Rica

**Keywords:** reconsolidation, fear memory, *Souroubea sympetala*, *Sin Susto*, betulinic acid, betulin

## Abstract

**Background:** *Souroubea sympetala* Gilg. is a neotropical vine native to Central America, investigated as part of a targeted study of the plant family Marcgraviaceae. Our previous research showed that extract of *S. sympetala* leaf and small branch extract had anxiolytic effects in animals and acts as an agonist for the GABA_A_ receptor at the benzodiazepine binding site. To date, the potential effects of *S. sympetala* and its constituents on reconsolidation have not been assessed. Reconsolidation, the process by which formed memories are rendered labile and susceptible to change, may offer a window of opportunity for pharmacological manipulation of learned fear. Here, we assessed the effects of *S. sympetala* crude extract and isolated phytochemicals (orally administered) on the reconsolidation of conditioned fear. In addition, we explored whether betulin (BE), a closely related molecule to betulinic acid (BA, an active principal component of *S. sympetala*), has effects on reconsolidation of learned fear and whether BE may synergize with BA to enhance attenuation of learned fear.

**Method:** Male Sprague–Dawley rats received six 1.0-mA continuous foot shocks (contextual training). Twenty-four hours later, rats were re-exposed to the context (but in the absence of foot shocks). Immediately following memory retrieval (recall), rats received oral administration of *S. sympetala* extract at various doses (8–75 mg/kg) or diazepam (1 mg/kg). In separate experiments, we compared the effects of BA (2 mg/kg), BE (2 mg/kg), and BA + BE (2 mg/kg BA + 2 mg/kg BE). The freezing response was assessed either 24 h later (day 3) or 5 days later (day 7). Effects of phytochemicals on fear expression were also explored using the elevated plus maze paradigm.

**Results:** *S. sympetala* leaf extract significantly attenuated the reconsolidation of contextual fear at the 25- and 75-mg/kg doses, but not at the 8-mg/kg dose. Furthermore, BA + BE, but not BA or BE alone, attenuated the reconsolidation of learned fear and exerted an anxiolytic-like effect on fear expression.

## Introduction

Fear-based disorders such as anxiety and posttraumatic stress disorder (PTSD) make up a significant portion of all diagnosed psychiatric conditions. A recent large-scale study found that among college students worldwide, major depression and anxious disorders are the most prevalent—with generalized anxiety and panic disorders constituting a combined 23.6% of all psychiatric conditions (with a 12-month prevalence rate for all mental conditions among the sampled population of over 30%; Auerbach et al., 2018). Despite that they are relatively commonplace, existing treatments for fear-based disorders are extremely limited. Pharmacological interventions for generalized anxiety are largely limited to benzodiazepines, while serotonergic–noradrenergic reuptake inhibitors (SNRIs) are also in use for panic disorder.

Moreover, although PTSD affects a large percentage of individuals following exposure to trauma (Van Ameringen et al., 2008), there are no specific pharmacological treatments targeting PTSD. Rather, the current treatments instead aim to treat the comorbid symptoms of anxiety and/or depression (Morina et al., 2014). It is also of interest to note that more than half of individuals with fear-based disorders seek alternative medicine (approaches to the treatment of illness which fall outside of the scope of standard medical care; Kessler et al., 2001). However, only 20% of those in the same study sought the guidance of a health care practitioner (i.e., a large percentage self-medicate; Kessler et al., 2001). As a result, it is clear that new pharmacotherapies are needed to specifically address PTSD (as well as other fear-based disorders).

**Figure 1:**
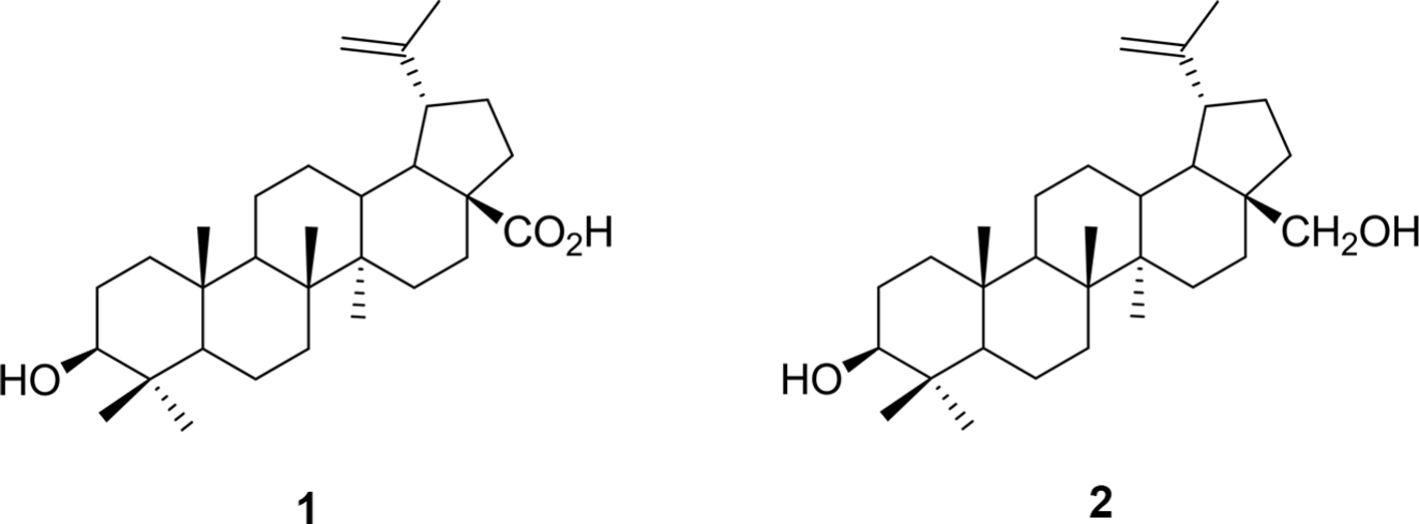
Chemical structure of (*1*) betulinic acid and (*2*) betulin.

In an attempt to discover alternate treatment strategies, efforts have been underway to explore plant-based remedies. Indeed, some plant extracts have been shown to exert anxiolytic-like effects (Arnason et al., 1981; Bourbonnais-Spear et al., 2006; Bourbonnais-Spear et al., 2007; Galdino et al., 2012; Bahi et al., 2014), and in particular we found that a Canadian genotype, *Sedum roseum* (L.) Scop. (syn. *Rhodiola rosea* Murkar et al., 2016), may be useful for the development of novel therapeutic interventions for human disorders based in fear memory. As part of our collaborative research to find new anxiolytics, we targeted the poorly studied family of neotropical vines, the Marcgraviaceae, in Central America. In preclinical tests modeling anxiety-like symptoms in animals, extract of one of these vines, *Souroubea sympetala* Gilg. (Marcgraviaceae; *Sin Susto*), has shown promise as an effective anxiolytic. Betulinic acid (BA; the most prominent triterpenoid constituent of the plant) is one of the active molecules responsible for this effect (see **Figure 1**; Mullally et al., 2011; Puniani et al., 2015). Both *S. sympetala* leaf extract and betulinic acid reduced stressor-induced cortisol secretion in rainbow trout and canines (Mullally et al., 2017; Masic et al., 2018). Evidence also suggests *S. sympetala* and BA may exert their effects on fear learning and expression by agonizing the benzodiazepine (BZD) binding site of the GABA_A_ receptor (Mullally et al., 2014). This is in line with evidence suggesting that anxiolytic botanicals frequently possess agonistic qualities for the GABAergic system (Awad et al., 2009).

In contrast to traditional GABA_A_ BZD receptor agonists such as diazepam, our previous research with *S. sympetala* leaf extract indicated that there were no withdrawal effects in rodents (Cayer, 2011). Thus, the plant might effectively modulate learned fear and/or anxiety with limited negative side effects typically associated with GABA_A_ BZD agonists. *S. sympetala* extracts and its combinations with another BA-producing plant have been safe in 30-day feeding trials at elevated doses with dogs (Villalobos et al., 2014; Liu et al., 2017).

In addition to its experimentally demonstrated activity as an anxiolytic, *S. sympetala* may also possess memory-altering qualities that prove useful for the treatment of other human mood disorders. Reconsolidation blockade, a technique for pharmacologically altering formed memories, may provide an avenue for developing novel treatment approaches for alleviating traumatic memories which are resistant to extinction. Evidence suggests that following retrieval, memories may return to a labile state in which they are susceptible to manipulation (Nader et al., 2000). During this period (the reconsolidation window), pharmacological disruption of memory re-stabilization can potentially diminish conditioned fear responses (and thus their expression). This technique has been used in preclinical research to help identify potential therapeutic targets (Debiec and LeDoux, 2006; Lin et al., 2006; Pitman et al., 2011; Murkar et al., 2017). Some, such as the β-adrenergic antagonist propranolol, xenon (which blocks NMDA receptors), and gastrin-releasing peptide (homologue of the amphibian peptide bombesin), show promise of attenuating learned fear when administered during the reconsolidation window in rodents (Debiec and LeDoux, 2006; Meloni et al., 2014; Murkar et al., 2017). Although it has only been sparsely tested in humans, there is some evidence from translational studies to suggest that the blockade of reconsolidation may have practical applications. Propranolol, for example, reduces physiologic responses to traumatic imagery in sufferers of PTSD when administered post-recall (Brunet et al., 2008). However, findings are mixed, and subsequent follow-up studies have failed to replicate this effect (Spring et al., 2015). Thus, new targeted approaches are required to help identify viable treatments to attenuate fear memory reconsolidation in humans.

Since some phytochemicals have been shown to affect reconsolidation and expression of learned fear (Stern et al., 2012; Murkar et al., 2016), phytotherapy may offer a novel approach to reconsolidation blockade as a treatment paradigm for fear-based disorders. Phytochemicals that affect consolidation and/or extinction of fear memory, as well as compounds acting through GABA that attenuate fear expression, may have memory-altering properties that generalize to other processes such as reconsolidation (although reconsolidation and extinction are thought to be distinct processes at the cellular and molecular level; Suzuki et al., 2004). Research has demonstrated that reconsolidation of learned fear can be altered by GABAergic neurotransmission in rodents following memory recall (Bustos et al., 2009). As possible GABA_A_ BZD receptor agonists, *S. sympetala* extract and its active components might therefore be of use to the development of novel treatment approaches for fear-based disorders by affecting fear memory.

Here, we aimed to determine whether *S. sympetala* extract and its primary active component BA could block the reconsolidation of conditioned fear. In addition to BA and *S. sympetala* leaf and small branch extract (SIN), we also explored the effects of a more abundant but closely related molecule—betulin (BE)—on reconsolidation of conditioned fear, and also a combination of the two (BA + BE) to determine whether the effects of BA are altered by the addition of BE. Previous work has shown that amyrins of similar chemical structure synergize with BA to enhance its effects (Cayer, 2011). We hypothesized that a similar effect may be observed with BE, given its very similar structure to BA (BA is structurally identical to BE, except for the exchange of the alcohol group with a carboxylic acid group). We also hypothesized that the leaf and small branch extract and BA would significantly attenuate reconsolidation of conditioned fear in a dose-dependent manner and that the effects of BA would be amplified by co-administration with BE.

Finally, in order to test for more general effects of isolated compounds from SIN on fear expression and anxiety-like behavior, we explored the effects of isolated phytochemicals BE, BA, and a combination of the two using the elevated plus maze paradigm.

## Methods

### Animals

Male Sprague–Dawley rats (Charles River Laboratories International, Inc.; 180–200 g on arrival) were doubly housed and maintained on a 12-h light/dark cycle (lights on at 0700 hours). Temperature was maintained at 23°C and relative humidity at 37%. Throughout the duration of the study, animals had free access to food and water. All experiments were conducted in accordance with the guidelines established by the Canadian Council on Animal Care and approved by the University of Ottawa Animal Care Committee.

### Drugs and Injections

Animals were habituated to administration of oral sweetened condensed milk (vehicle) 1 week prior to the beginning of the experiment. Since evidence suggests *S. sympetala* extract may act as a GABA_A_ BZD receptor agonist, diazepam (Sandoz, Canada) was used as a positive control. Rats were randomly assigned to one of the treatment groups used throughout the experiments. Drug groups among all experiments consisted of 1) *S. sympetala* leaf and small branch extract (SIN, 8–75 mg/kg); 2) diazepam (positive control, 1 mg/kg); 3) BA (2 mg/kg); 4) BE (2 mg/kg); 5) BA + BE (2 mg/kg BA + 2 mg/kg BE); and 6) vehicle.

### Plant Extract, Isolated Phytochemicals, and Analysis

*S. sympetala* Gilg. leaves and small branches were originally collected in Tortuguero, Costa Rica, and propagated at a Universidad Nacional field in Sarapiqui. Samples were dried overnight in a commercial plant drier at 35°C. A voucher specimen was identified by two of us (MOR and PS) and deposited in the JVR herbarium, (see **Supplementary Figure 1**) Universidad Nacional Costa Rica (voucher no. 13231, in *Supplementary Data*). *S. sympetala* is an accepted name on the plant.list.org and tropicos.org. Plant material of *S. sympetala* was ground with a Wiley Mill (2-mm mesh size). Samples were incubated with shaking in 1:20 (weight/volume) ethyl acetate (EtOAc) for 12–15 h at room temperature. The solvent was filtered (Whatman no. 1) and the filter cake re-extracted twice with half as much EtOAc (1:10 and 1:5). The total solvent from the three extractions were combined for an exhaustive extraction. The solvent was removed *via* rotary evaporation with a Yamato Rotary Evaporator RE50 (Yamato Scientific, Japan) at 40°C, lyophilized (Super Modulyo, Thermo Electron, USA), and stored in opaque glass vials at 4°C. Analysis of the plant was undertaken using a validated high-performance liquid chromatography–mass spectrometry (HPLC-MS) method (Liu et al, 2019) which shows chromatograms for the analysis. The extract contained a mean (SE) of betulinic acid of 6.8 mg (0.2). Isolation, purification, and spectroscopic identification of betulinic acid and betulin have been described previously (Puniani et al., 2015) and was found to be greater than 95% pure by HPLC-MS.

### Contextual Fear Conditioning

The conditioning chambers (Coulbourn Instruments) measured 31 × 25 × 30 cm. The front and back walls were made of clear acrylic, and the two sidewalls and top made of stainless steel. The floor comprised 16 stainless steel bars (4-mm diameter spaced 1.4 cm apart) connected to Coulbourn precision-regulated animal shockers, which delivered scrambled foot shock (1.0 mA). Animals (*N* = 7–10/group) were randomly distributed into treatment groups. Subjects that failed to achieve a minimum baseline freezing level of 40% during recall (assessed on day 2) were removed from the analyses.

### Experimental Procedure

#### Experiment 1: Effects of Plant Extracts on Reconsolidation Blockade, Short Term

Animals were exposed to six consecutive 1-s foot shocks over the course of 11 min. Contextual conditioning was used (pairing of foot shock with the conditioning chamber). Twenty-four hours later, animals were re-exposed to the context in which they received the foot shock (conditioning chamber) for 5 min and freezing (total time spent in complete immobility) was measured (day 2, recall). Cage placement and assignment to drug treatment groups was randomized/counterbalanced.

**Figure 2:**
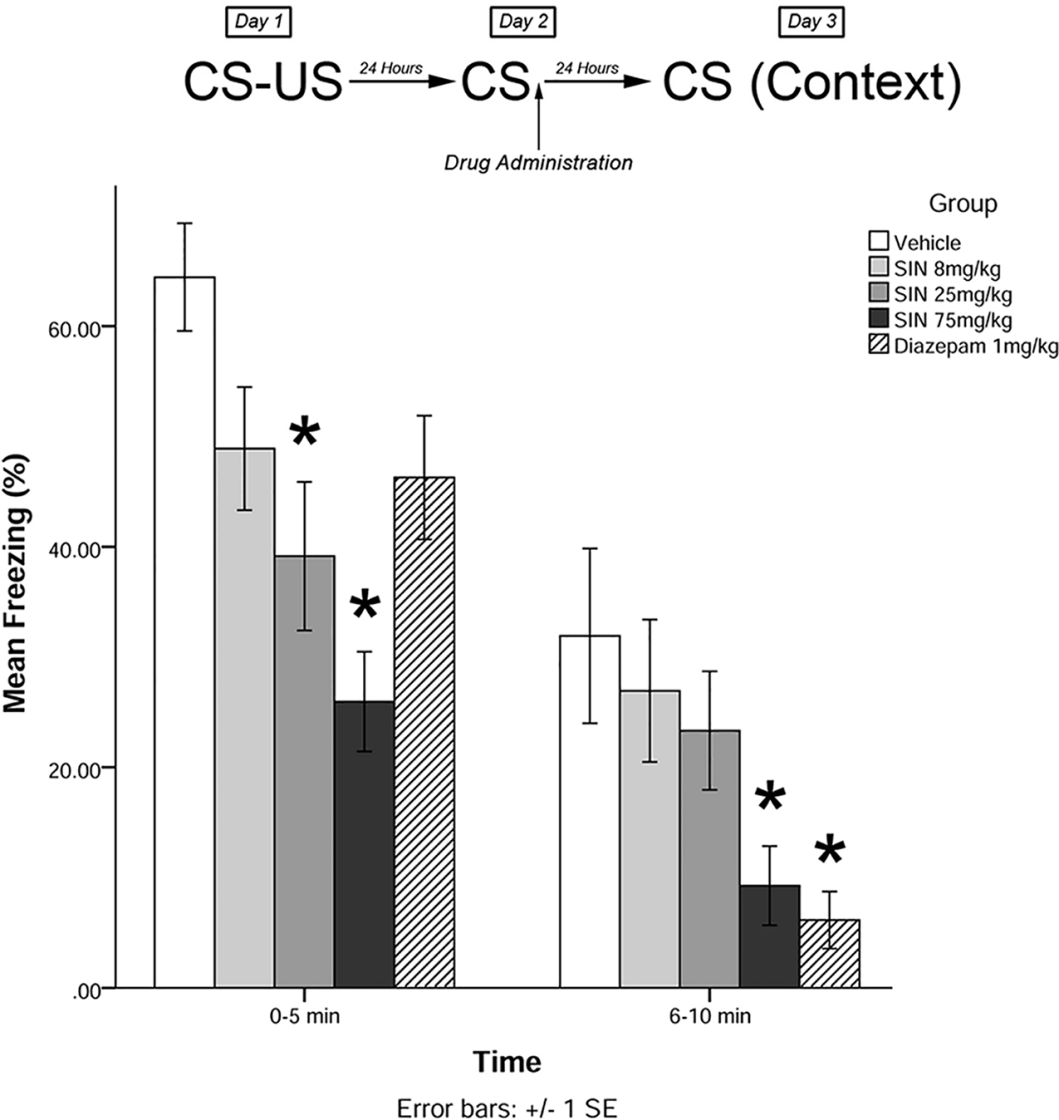
*S. sympetala* extracts attenuated the freezing response. 25 mg/kg SIN (approximate BA content 0.37 μmol/kg) and 75 mg/kg SIN (approximate BA content 1.12 μmol/kg), but not 8 mg/kg SIN (approximate BA content 0.12 μmol/kg), attenuated freezing on day 3 when administered immediately post-recall. * indicates *p* < 0.05.

Immediately after the 5-min recall session on day 2, animals were administered drugs in one of five treatment groups: 8 mg/kg plant extract (low dose), 25 mg/kg plant extract (medium dose), 75 mg/kg plant extract (high dose), vehicle, and 1 mg/kg diazepam (positive control). Twenty-four hours later (day 3), animals were re-exposed to the conditioning chamber and freezing was measured over the course of 10 min in blocks of 0–5 and 6–10 min. The timeline of procedures for experiment 1 is illustrated in **Figure 2**.

#### Experiment 2: Long-Term Effects on Reconsolidation Blockade

**Figure 3:**
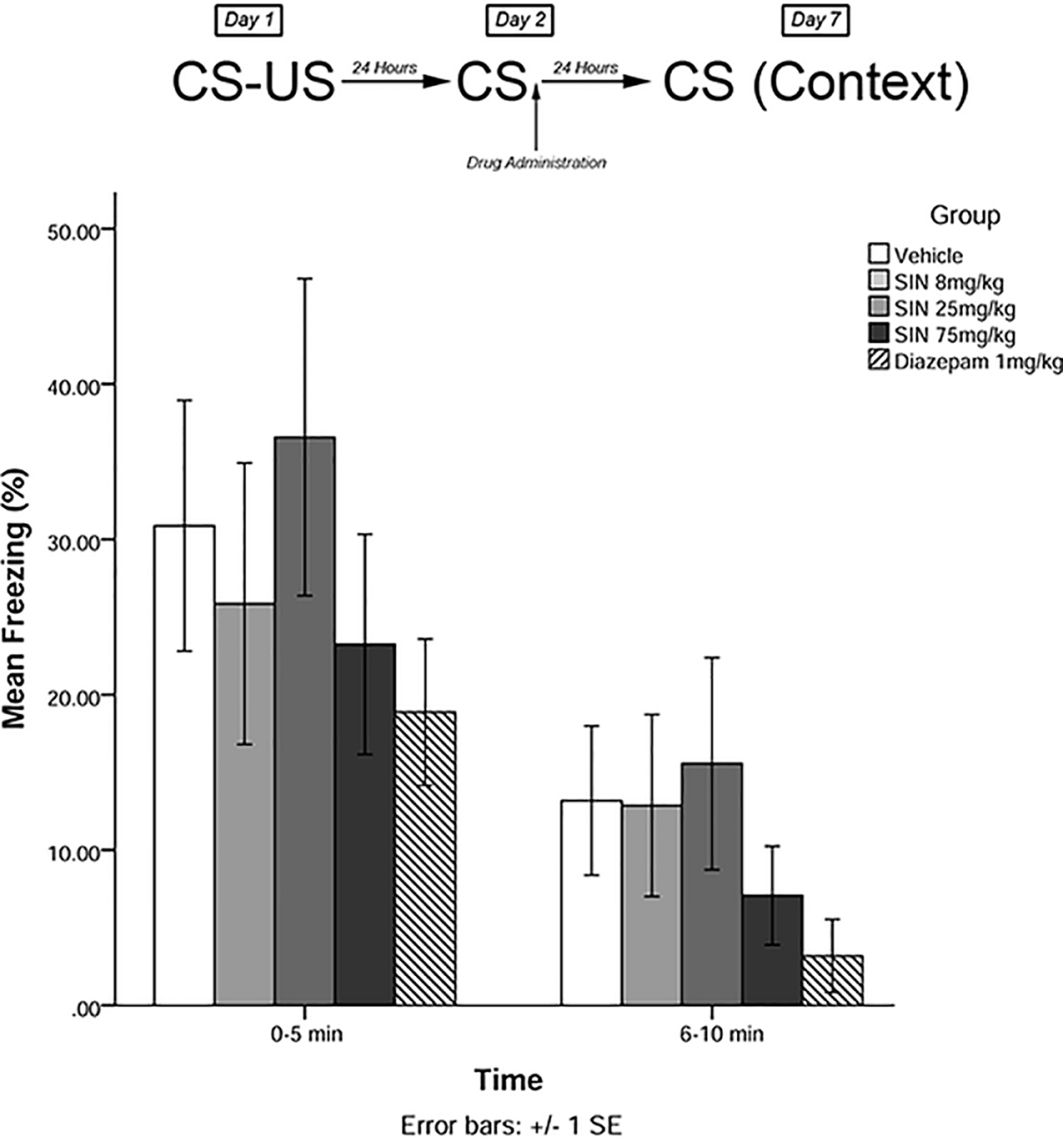
*S. sympetala* extract administered immediately following recall did not attenuate freezing on day 7.

The same training procedures as experiment 1 were utilized to test for long-term effects of plant compounds on fear memory reconsolidation blockade. Immediately after the 5-min recall session on day 2, animals were administered drugs in one of five treatment groups: 8 mg/kg plant extract (low dose), 25 mg/kg plant extract (medium dose), 75 mg/kg plant extract (high dose), vehicle, and 1 mg/kg diazepam (positive control). Five days later (day 7), animals were re-exposed to the conditioning chamber and freezing was measured over the course of 10 min in blocks of 0–5 and 6–10 min. The timeline of procedures for experiment 2 is illustrated in **Figure 3**.

#### Experiment 3: Short-Term Effects of Plant Extracts on Reconsolidation Blockade

**Figure 4:**
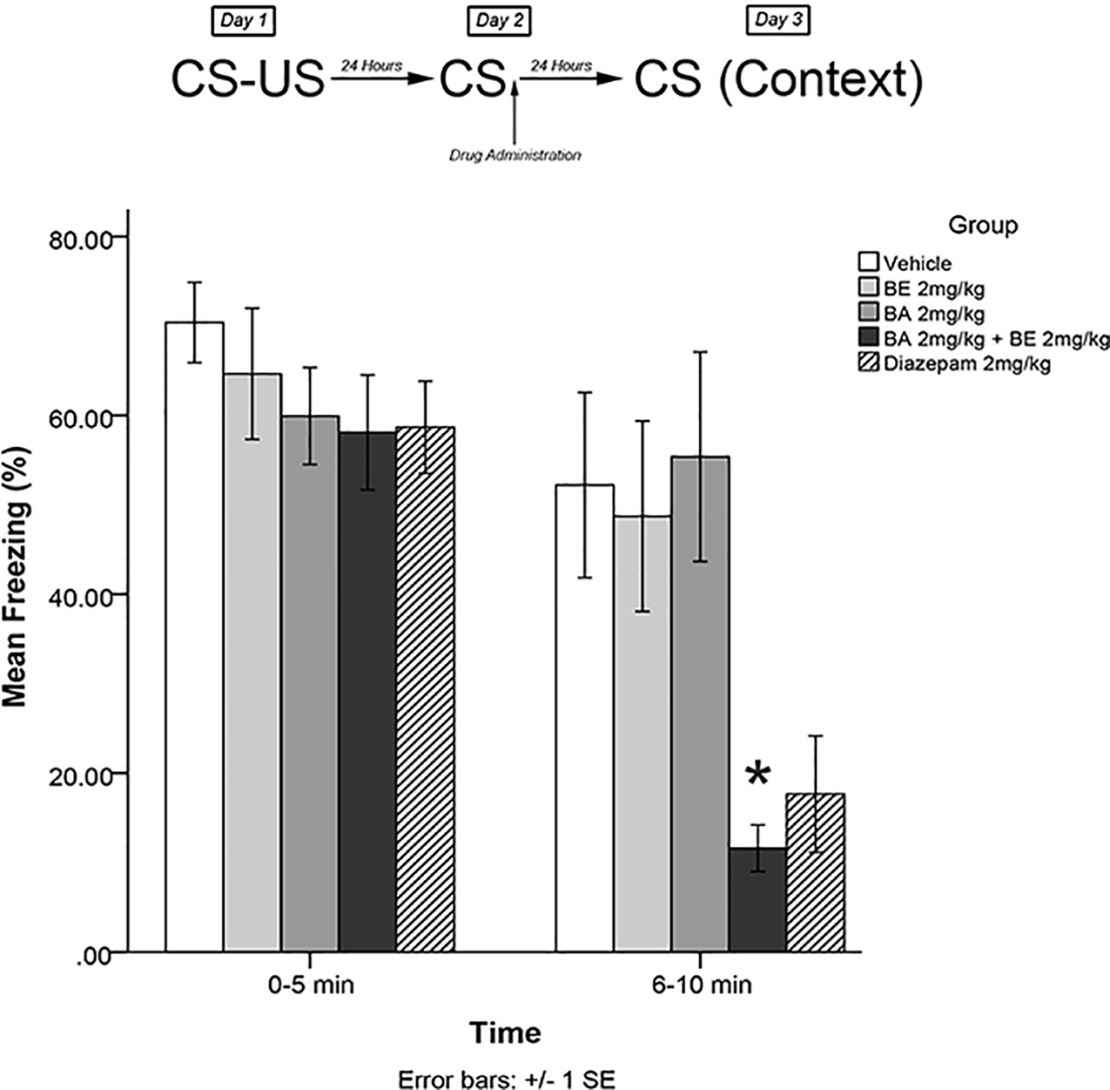
Isolated phytochemicals betulinic Acid (BA) 2 mg/kg (4.38 μmol/kg) and betulin (BE) 2 mg/kg (4.52 μmol/kg) attenuated reconsolidation of conditioned fear when co-administered, but not alone. The combined dose of BA + BE more closely resembles *S. sympetala* extract. * indicates p < 0.05.

The same training procedures as experiment 1 were utilized to test for short-term effects of isolated plant compounds on fear memory reconsolidation blockade. Immediately after the 5-min recall session on day 2, animals were administered drugs in one of four treatment groups: 2 mg/kg BE, 2 mg/kg BA, 2 mg/kg BE + 2 mg/kg BA, or vehicle. Twenty-four hours later (day 3), animals were re-exposed to the conditioning chamber and freezing was measured over the course of 10 min in blocks of 0–5 and 6–10 min. The timeline of procedures for experiment 3 is illustrated in **Figure 4**.

#### Experiment 4: Long-Term Effects of Plant Extracts on Reconsolidation Blockade

**Figure 5:**
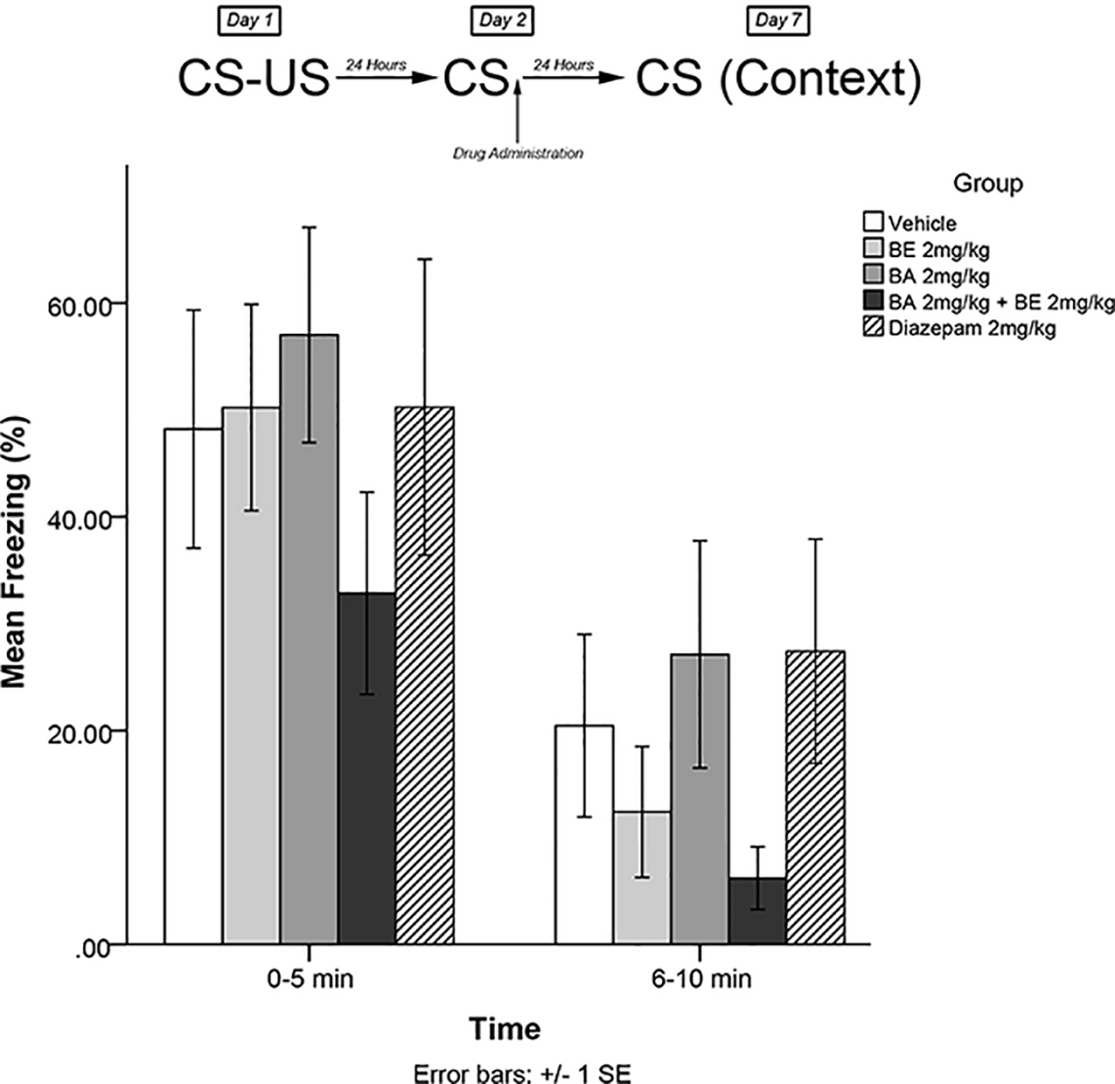
Isolated phytochemicals 2 mg/kg (4.52 μmol/kg) BE and 2 mg/kg (4.38 μmol/kg) BA did not attenuate freezing on day 7.

The same training procedures as experiment 1 were utilized to test for long-term effects of plant compounds on fear memory reconsolidation blockade. Immediately after the 5-min recall session on day 2, animals were administered drugs in one of four treatment groups: 2 mg/kg BE, 2 mg/kg BA, 2 mg/kg BE + 2 mg/kg BA, or vehicle. Five days later (day 7), animals were re-exposed to the conditioning chamber and freezing was measured over the course of 10 min in blocks of 0–5 and 6–10 min. The timeline of procedures for experiment 4 is illustrated in **Figure 5**.

#### Experiment 5: Attenuation of the Fear Response Requires Reactivation of the Fearful Memory Trace

**Figure 6:**
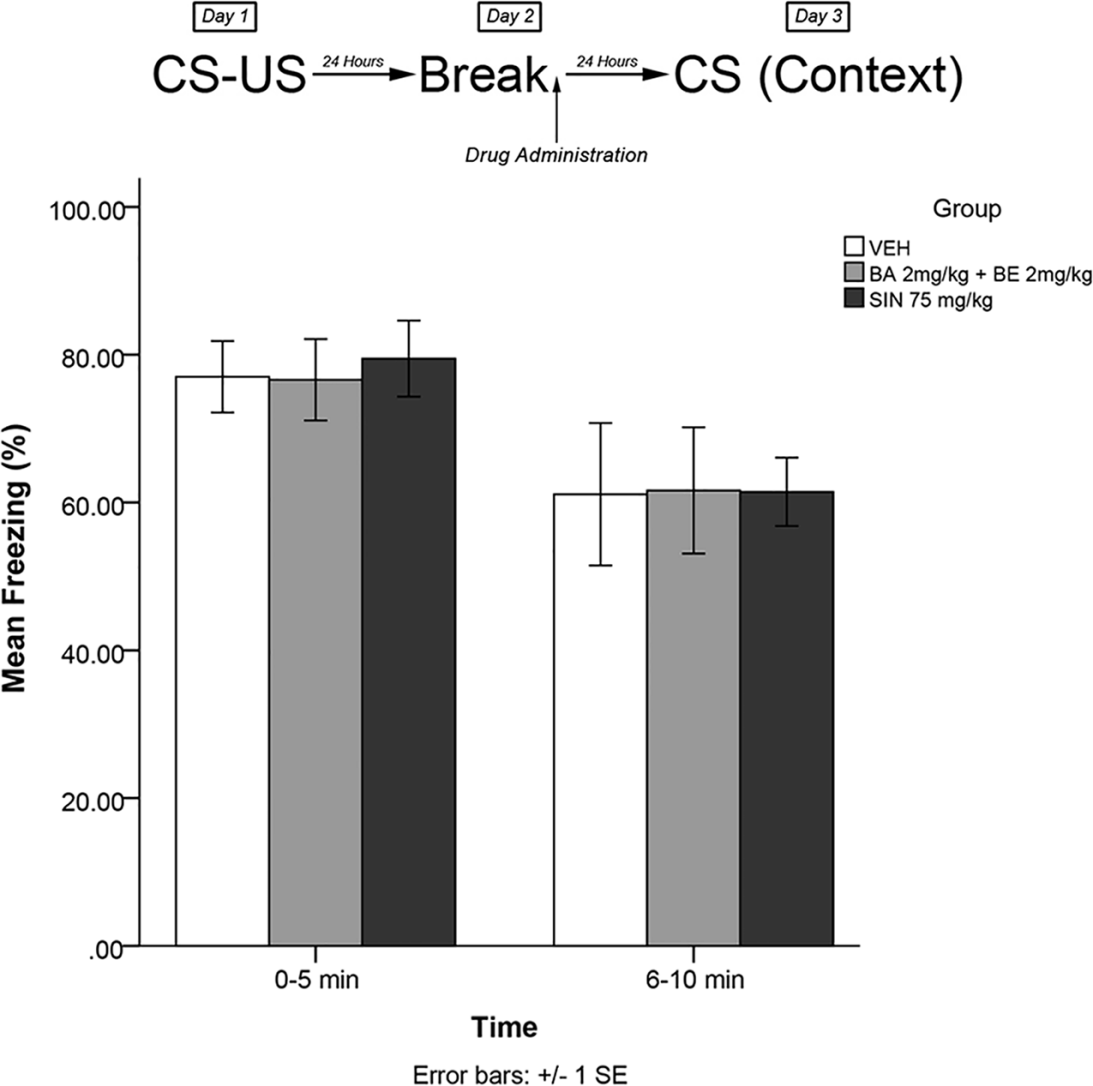
SIN 75 mg/kg (approximate BA content 1.12 μmol/kg) and a combined dose of 2 mg/kg (4.52 μmol/kg) BE + 2mg/kg (4.38 μmol/kg) BA did not produce attenuation of the learned fear response when administered in the absence of re-exposure to the fearful stimulus.

In order to determine whether the effects were dependent upon memory trace reactivation, an experiment was performed using identical procedures to experiment 1 and experiment 3. In this experiment, however, animals were not re-exposed to the fearful stimulus on day 2. The timeline of procedures for experiment 5 is illustrated in **Figure 6**.

#### Experiment 6: Elevated Plus Maze

The elevated plus maze (EPM), a paradigm used to characterize anxiety-like behavior, was conducted in accordance with methods as described by Cayer et al. (2013). The maze consisted of two open arms (50 × 10 cm) and two perpendicular arms enclosed by high walls (40 cm tall). The maze was placed 50 cm above the ground. Percentage of time spent in the open arms and number of unprotected head dips were measured.

### Statistical Analyses

All statistical analyses were conducted using IBM Statistics Package for the Social Sciences^®^ (SPSS) 20. Data was analyzed using mixed-measures ANOVA, in which drug treatment was the between -groups variable and time was the within-groups variable. Greenhouse–Geisser correction was applied where the assumption of sphericity is violated. Follow-up comparisons of significant main effects and interaction effects were conducted using Bonferroni-corrected *t*-tests, or corrected *post hoc* tests where the assumption of homogeneity of variance was violated.

## Results

### Experiment 1

The mixed-measures ANOVA revealed a significant main effect of treatment on levels of freezing [*F*(4,41) = 5.523, *p* < 0.01]. Levene’s test revealed the assumption of equality of error variances was violated at 6–10 min (*p* < 0.05). There was also a significant time by group interaction effect [*F*(4,41) = 4.485, *p* < 0.05].

*Post hoc* analyses further revealed that animals who received medium and high doses of plant extract immediately following memory recall on day 2 displayed significantly less freezing than vehicle-treated animals on day 3 at 0–5 min (*p* < 0.05; see **Figure 2**). Animals that received high-dose plant extract or diazepam immediately following recall on day 2 also displayed significantly less freezing than vehicle-treated animals on day 3 at 6–10 min (*p* < 0.05).

### Experiment 2

The mixed-measures ANOVA revealed no significant main effects or interaction effects [*F*(4,41) = 0.291, *p* > 0.05] (see **Figure 3**).

### Experiment 3

The mixed-measures ANOVA revealed a significant time by group interaction effect [*F*(4,40) = 5.227, *p* < 0.01] and a significant main effect of group [*F*(4,40) = 3.505, *p* < 0.05]. Levene’s test revealed the assumption of equality of error variances was violated [*F*(4,40) = 4.549, *p* < 0.05]. Games–Howell corrected *post hoc* analysis revealed that rodents that had received BA 2 mg/kg + BE 2 mg/kg exhibited significantly less freezing than vehicle-treated animals (*p* < 0.05) at the 6- to 10-min interval (see **Figure 4**). No other groups significantly differed from vehicle at either the 0- to 5- or 5- to 10-min interval (all *p* > 0.05).

### Experiment 4

The mixed-measures ANOVA revealed no significant main effects or interaction effects, [*F*(4,40) = 0.952, *p* > 0.05] (see **Figure 5**).

### Experiment 5

The mixed-measures ANOVA revealed no significant main effects or interaction effects, [*F*(2,27) = 0.062, *p >* 0.05] (see **Figure 6**).

### Experiment 6

**Figure 7:**
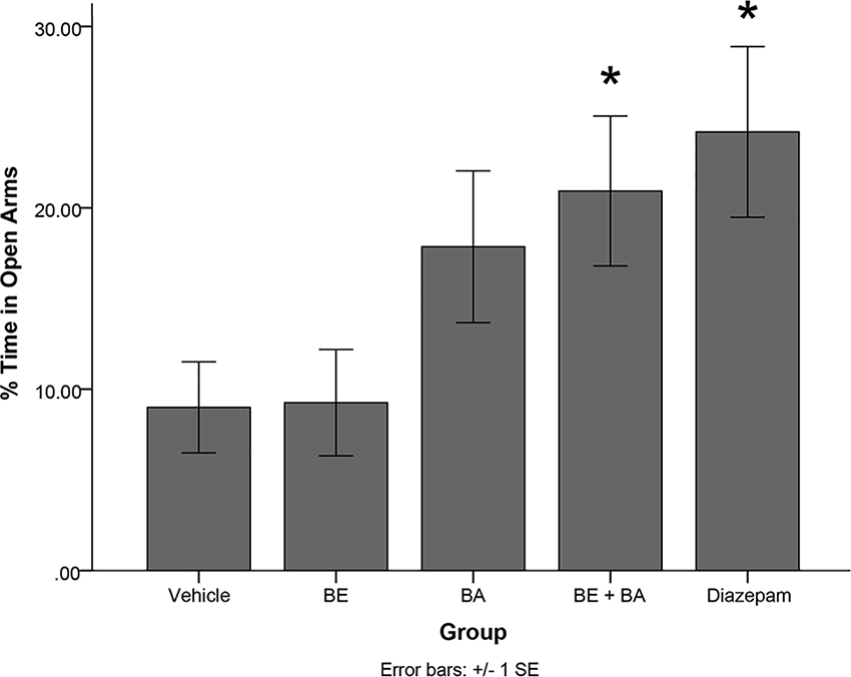
Rodents treated with 2 mg/kg (4.52 μmol/kg) BE + 2 mg/kg (4.38 μmol/kg) BA, as well as 1 mg/kg (3.51 μmol/kg) diazepam, exhibited significantly more time in the open arms of the maze than rodents treated with vehicle. * indicates p < 0.05.

A one-way ANOVA revealed a significant main effect of treatment group in the EPM paradigm on percentage of time spent in the open arms of the maze [*F*(4,40) = 3.297, *p* < 0.05]. Levene’s test indicated the assumption of equality of error variances was not violated (*p >* 0.05). Follow-up analyses indicated that rats treated with diazepam (*p <* 0.05) and a combination of BA + BE (2 mg/kg each; *p* < 0.05) spent significantly more time in the open arms of the EPM than vehicle-treated animals (see **Figure 7**). No other treatment groups significantly differed from vehicle.

**Figure 8:**
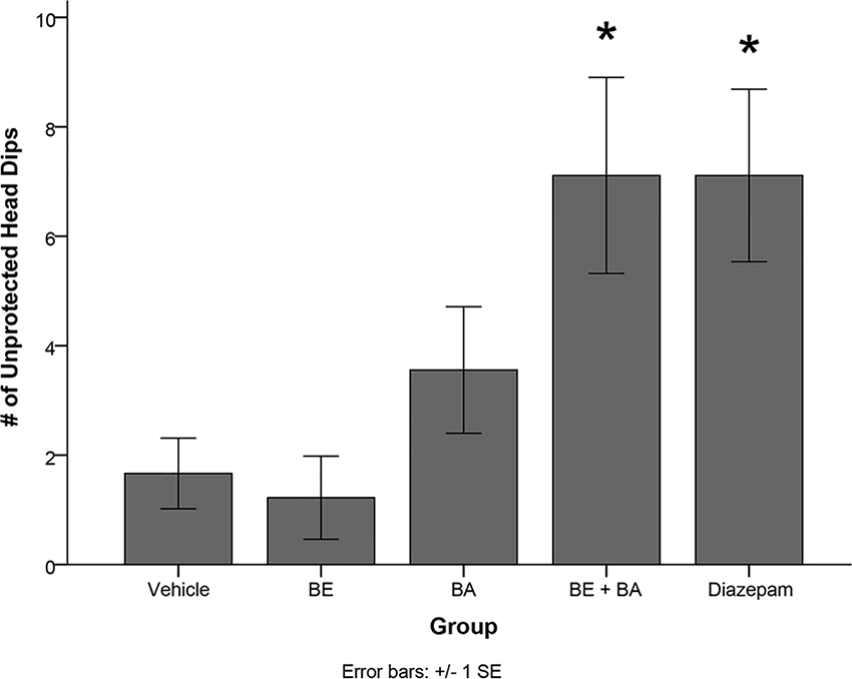
Rodents treated with 2 mg/kg (4.52 μmol/kg) BE + 2 mg/kg (4.38 μmol/kg) BA, as well as 1 mg/kg (3.51 μmol/kg) diazepam, exhibited significantly more unprotected head dips than rodents treated with vehicle. * indicates p < 0.05.

One-way ANOVA also revealed a significant treatment effect on the number of unprotected head dips [*F*(4,40) = 5.084, *p* < 0.01]. Levene’s test indicated the assumption of equality of error variances was violated (*p* > 0.05). Follow-up analyses indicated that rats treated with BA + BE (*p <* 0.05) and diazepam (*p* < 0.05) had significantly more unprotected head dips than vehicle-treated animals (see **Figure 8**). No other groups significantly differed from vehicle.

## Discussion

Our findings suggest that *S. sympetala* leaf and small branch extract is effective in modulating reconsolidation of learned fear and fear expression. In experiment 1, 25 mg/kg SIN and 75 mg/kg SIN (but not 8 mg/kg SIN) effectively attenuated the learned fear response as measured on day 3 when administered immediately post-recall. This suggests that *S. sympetala* plant extract may have therapeutic potential for modulating reconsolidation of learned fear. However, there was no significant effect of SIN at day 7 (long-term testing).

Our findings also demonstrated significant effects of 2 mg/kg BA + 2 mg/kg BE both on reconsolidation of learned fear responses and on fear expression in the EPM paradigm. However, similarly to what we observed with SIN, BA + BE was effective at attenuating reconsolidation of learned fear in the short term, but not the long term. Although it is possible the effects of SIN and BA + BE are not permanent, this would be inconsistent with previous work suggesting that memories targeted by reconsolidation blockade are not prone to reinstatement (Nader et al., 2000; Bustos et al., 2009; Meloni et al., 2014; Murkar et al., 2017). Rather, we anticipate that a floor effect is responsible for this observation in both cases since mean freezing levels at day 7 in both long-term experiments were much lower overall for all groups in both of our long-term experiments. A reduction in freezing levels with the CER paradigm after 7 days is unfortunately not unusual. Future work should therefore aim to determine whether the effects of SIN and its active principal components persist in the long term using measures that are less sensitive to drift over time and floor effects (e.g., fear-potentiated startle, which produces robust measurable effects even after longer periods). It would also be advantageous for future studies to include testing at a longer interval (i.e., day 10 or 15).

In addition to highlighting that SIN and BA + BE may effectively modulate learned fear and fear expression, our findings also suggest that BE may indeed synergize the effects of BA—since both BA and BE alone were ineffective at attenuating the learned fear response or fear expression, but a combined dose yielded significant effects on both reconsolidation of learned fear (in experiments 3) and fear expression (in experiment 6). The results of these experiments are in partial agreement with previous findings. For example, previously, our group showed that BA exerted an anxiolytic effect when administered alone (Puniani et al., 2015).

However, our prior work suggests that while BA may be an active principal of *S. sympetala* extracts, it is likely not the only active component. Thus, our combined BA + BE group may be the closest representation of the extract and the original leaf/small branch decoction *Sin Susto*, which contains both BA and a host of likely bioactive molecules.

Our findings here highlight the potential therapeutic benefit of *S. sympetala*, betulinic acid (BA), and betulin (BE, as a possible synergist) for mediation of learned fear and fear expression. However, further research is necessary to explore the nature of triterpenoid synergism. Future studies may benefit from a more extensive dose–response characterization of BA effects to determine more precisely the effective dose range of BA. In addition, although we have observed robust effects on behavior and memory, our experiments did not probe the central mechanisms mediating the effects of these compounds. Future studies should therefore aim to elucidate the central mechanisms by which *S. sympetala* extracts might exert their effects. Since evidence suggests BA and *S. sympetala* extract may act as GABA_A_ BZD receptor agonists (Mullally et al., 2014), it would be an important future step to determine whether the effects of orally administered SIN and/or BA + BE are mediated centrally and whether these effects can be blocked by a selective GABA_A_ BZD binding site antagonist (such as flumazenil) as well as with site-specific microinjection of those antagonists at brain regions where GABAergic transmission is known to play a role in learned fear (e.g., the basolateral complex of the amygdala; Duvarci and Pare, 2014).

Overall, our data suggests that *S. sympetala* extracts and isolated phytochemicals may prove useful as pharmacological targets for the development of new and more effective treatments for human fear-based disorders such as PTSD. Furthermore, in contrast to the current mainline treatments for anxiety, some experiments suggest that BA produced no changes in weight gain or locomotor activity, as well as no withdrawal effects on food intake, fecal output, and a variety of light-phase and dark-phase behaviors including scratching, grooming, resting, and exploring (Cayer, 2011) while maintaining fulsome therapeutic effects on fear expression. Whether *S. sympetala* extracts produce withdrawal effects in humans after chronic administration, however, has yet to be explored. It will be a necessary and important step for future studies to determine whether—as suggested by its use in traditional native medicine—the effects of *S. sympetala* extracts translate well to studies with human participants.

## Data Availability Statement

The datasets generated for this study are available on request to the corresponding author.

## Ethics Statement

The animal study was reviewed by the University of Ottawa Animal Care Committee. All experiments were conducted in accordance with the guidelines established by the Canadian Council on Animal Care and approved by the University of Ottawa Animal Care Committee.

## Author Contributions

AM wrote the paper, analyzed data, performed experiments, and contributed to study design. JJ and CC performed experiments. ZM revised the paper and contributed to study design. TD, JA, PS-V, and MO produced plant materials, extracts, and pure compounds used in the experiments and contributed to study design.

## Funding

The experimental protocol for this study was approved by the University of Ottawa Animal Protocol Review Committee and conforms to the guidelines of the Canadian Council on Animal Care. This research was supported by a discovery grant that was awarded to ZM (application number 05388) from the Natural Sciences and Engineering Research Council of Canada (NSERC) and a Doctoral Research Award that was awarded to AM (application number 336752) from the Canadian Institutes of Health Research (CIHR).

## Conflict of Interest

ZM, TD, PS-V, and JA have direct involvement in Souroubea Botanicals Inc., which has brought the research to market in animal care field.

The remaining authors declare that the research was conducted in the absence of any commercial or financial relationships that could be construed as a potential conflict of interest.
