# Supplementary figures and images for: Extract and Active Principal of the Neotropical Vine Souroubea sympetala Gilg. Block Fear Memory Reconsolidation
Source: Front Pharmacol. 2019 Dec 20;10:1496. doi: 10.3389/fphar.2019.01496 (PMC6951415; doi:10.3389/fphar.2019.01496)

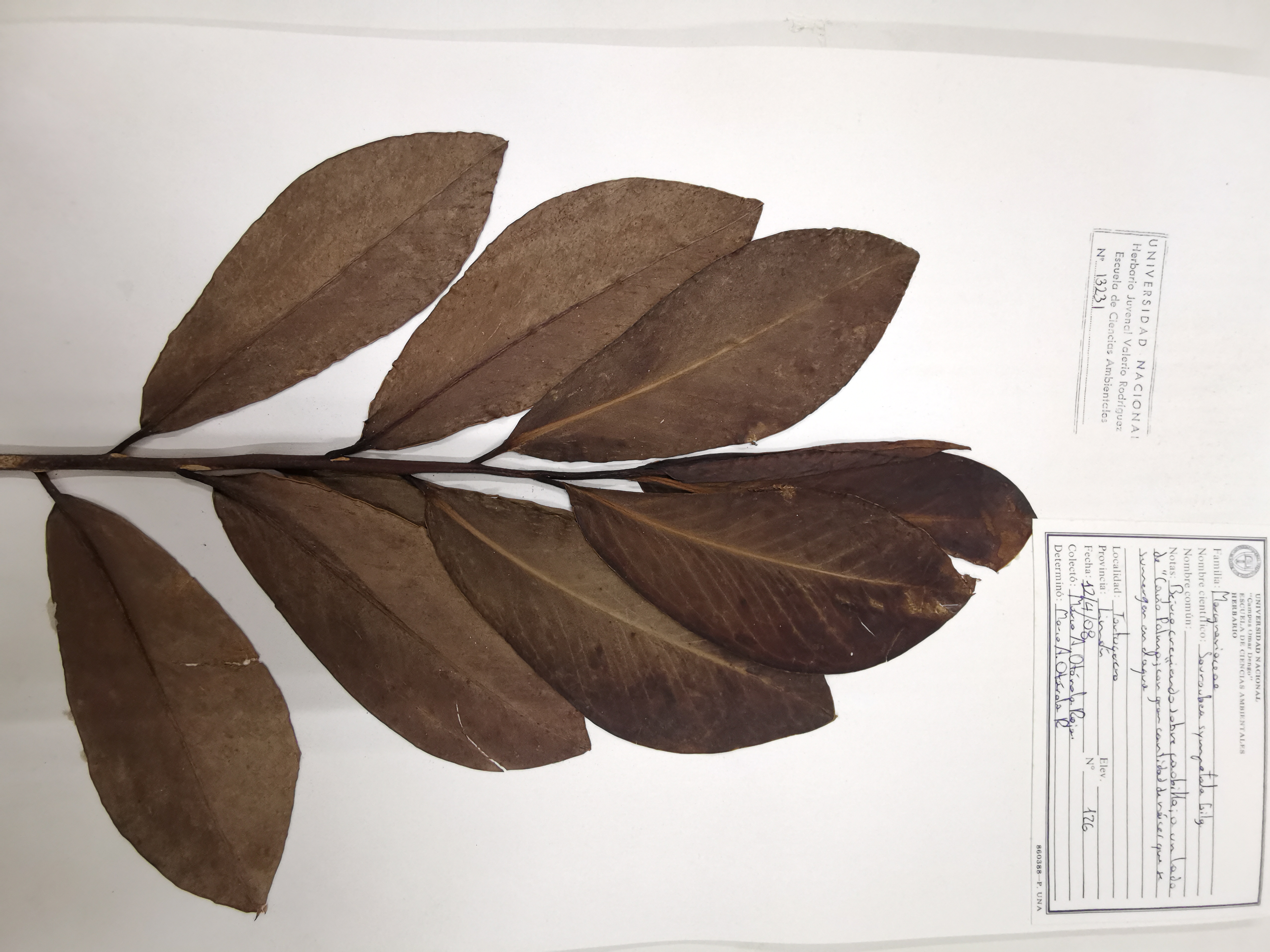

Supplement: Supplementary file 1 [file Image_1.jpeg]
